# The Metabolic Orchestration of Immune Evasion in Glioblastoma: From Molecular Perspectives to Therapeutic Vulnerabilities

**DOI:** 10.3390/cancers17111881

**Published:** 2025-06-04

**Authors:** Ravi Medikonda, Matthew Abikenari, Ethan Schonfeld, Michael Lim

**Affiliations:** Department of Neurosurgery, Stanford University School of Medicine, Stanford, CA 94304, USA; rmediko1@stanford.edu (R.M.); mattabi@stanford.edu (M.A.); ethan.schonfeld@stanford.edu (E.S.)

**Keywords:** glioblastoma, immunometabolism, tumor microenvironment, immune evasion, tryptophan metabolism, arginine depletion, sphingolipid pathways

## Abstract

Glioblastoma (GBM) is a highly aggressive primary brain cancer with dismal prognoses despite current standards of care. Immunotherapy is being explored for GBM, given its promising results in other solid malignancies; however, the results from early clinical studies in GBM are disappointing. It has been discovered that GBM has numerous mechanisms of immune resistance, including the physical blood–brain barrier, high intratumoral and intertumoral heterogeneity, and numerous cellular and molecular components in the tumor microenvironment (TME) that promote immunosuppression. Furthermore, GBM utilizes numerous metabolic pathways to establish a survival advantage in the TME. Recently, it has begun to become evident that these complex metabolic pathways that promote GBM growth and invasion also contribute to tumor immune resistance. Aerobic glycolysis provides tumor cells with ample ATP while depleting key glucose and increasing acidity in the TME. Increased glutamine, tryptophan, and arginine metabolism deprives T cells of these necessary amino acids for proper anti-tumor function. Sphingolipid metabolism promotes an immunosuppressive phenotype in the TME and affects immune cell trafficking. This review will discuss, in detail, the key metabolic pathways relevant to GBM pathophysiology which also modulate host immunosuppression.

## Simple Summary

Glioblastoma is a deadly cancer with poor outcomes attributed to numerous factors including a tremendous ability for the tumor to evade and suppress the body’s immune system. In recent years, it has been discovered that the same pathways that are responsible for GBM proliferation may also be contributing to the ability for this tumor to suppress the immune system. This review presents the key findings linking numerous critical pathways that sustain GBM growth to tumor immunosuppression.

## 1. Introduction

Glioblastoma (GBM) is an aggressive and treatment-refractory primary brain tumor characterized by rapid infiltrative growth, heterogeneity, and concerted resistance to immune-based therapies [1]. Historically, standards of care, including maximal safe resection, radiotherapy, and temozolomide chemotherapy, have resulted in a median survival of 15–16 months [2]. Newer treatment strategies such as tumor treatment fields (TTFs) are gradually becoming a part of GBM standard of care as we are starting to see an increase in median overall survival (mOS) [3,4]. For instance, Stupp et al. showed that TTFs plus temozolomide had an overall survival of 20.9 months compared to 16 months in the temozolomide-only arm [5]. Nevertheless, progress in improving GBM prognosis has been slow and, in the past, strategies to enhance immune system engagement such as immune checkpoint blockade have largely failed in GBM due to a poor understanding of the numerous mechanisms of GBM immune resistance and the profoundly immunosuppressive GBM tumor microenvironment (TME) [6,7,8].

An emerging paradigm in elucidating GBM immune resistance resides in better understanding the interplay between cancer metabolism and immunosuppression. Cancer metabolism was historically conceptualized in terms of the Warburg effect, whereby tumor cells preferentially utilize aerobic glycolysis for energy generation [9]. However, our understanding of cancer metabolism has expanded significantly in recent years. GBM induces a multi-axis metabolic reprogramming of its environment that directly promotes tumor survival and immune escape [10,11,12].

GBM cells utilize key metabolic substrates, including glucose, glutamine, and other amino acids, while also secreting immunomodulatory metabolic byproducts including lactate, kynurenine, and adenosine. Through depleting key metabolic substrates and secreting metabolic byproducts, GBM promotes immunosuppression by reprogramming immune cells, inducing T-cell exhaustion, and decreasing antigen presentation [13,14,15,16]. For instance, tumor-associated macrophages (TAMs) or myeloid-derived suppressor cells (MDSCs) reprogram into tolerogenic, M2-like phenotypes driven by lipid metabolism and lactate exposure [1,17,18]. In contrast, cytotoxic T cells face a functional lack of nutrients in a nutrient-poor environment, limiting their metabolic programs to maintain killing or persistence [19,20]. This metabolic bottleneck is compounded by oxygen stress occurring in hypoxic areas of tumors, which activates HIF-1α–driven gene programs that go beyond promoting angiogenesis and promote immunosuppressive accumulation of metabolites [21,22,23].

A defining feature of the GBM TME is the manifestation of metabolic survival of the fittest: GBM hoards nutrients and induces a nutrient scarcity in the TME in order to promote its own survival and simultaneously orchestrate immune evasion by making it difficult for immune cells to obtain the resources they need for survival. In this highly controlled and hostile niche, immune cells and tumor cells compete for important resources necessary for effector function, immune cell survival, and epigenetic programming. The outcome of this competition is rarely neutral; it is decisively in favor of tumor persistence and immune dysfunction.

This review summarizes our understanding of GBM immunometabolism with a focus on how several important metabolic pathways contribute to immunosuppression in GBM including glycolysis, amino acid depletion, and sphingolipid metabolism. Furthermore, this review will explore how hypoxic conditions in the TME also promote immunosuppression. The focus of this review is to highlight these key immunometabolic pathways in GBM and discuss the potential to exploit these mechanisms to devise novel and synergistic GBM treatment strategies.

## 2. Isocitrate Dehydrogenase Mutational Status Affects Glioma Classification

The significance of metabolic pathways to glioma pathophysiology is underscored by the isocitrate dehydrogenase-1 (IDH1) enzyme. The mutational status of this enzyme is utilized in GBM classification and distinguishes GBM from lower-grade glioma. The wildtype IDH1 enzyme catalyzes the conversion of isocitrate to α-ketoglutarate (α-KG) [24]. IDH plays a critical role in numerous biological processes, including ATP generation and the Krebs cycle, glutamine metabolism, and lipid synthesis [25]. Mutations in the IDH1 gene have been associated with numerous malignancies including lower-grade gliomas as the mutant IDH1 enzyme catalyzes the conversion of α-KG to D-2-hydroxyglutarate (D-2HG), an oncometabolite [26]. The accumulation of D-2HG in IDH-mutant gliomas contributes to cancer progression by inducing changes in cellular metabolism and epigenetic modifications [27,28]. IDH1 mutation status is an important molecular marker that has been used by the World Health Organization Classification of Tumors of the Central Nervous System to define GBM as IDH1-wildtype, and IDH-mutant gliomas are characterized as astrocytoma or oligodendroglioma [29]. The IDH mutation presents a therapeutic target in lower grade gliomas but not in primary GBM which possesses the wildtype IDH enzyme [30]. Given the WHO classification of gliomas, IDH mutational status has become an important prognostic marker that separates lower grade gliomas from high-grade GBM, which has a worse prognosis.

## 3. The Role of Glycolysis in GBM Immunosuppression

GBM cells possess a hyperactivated glycolytic phenotype supported by the overexpression of glucose transporters GLUT1 and GLUT3, as well as glycolytic enzymes such as hexokinase 2 (HK2), lactate dehydrogenase A (LDHA) and pyruvate dehydrogenase kinase 1 (PDK1) [31,32,33]. This metabolic phenotype rapidly consumes glucose in the TME to generate ATP and renders the TME glucose deficient. Glucose deficiency has a detrimental effect on invading CD8^+^ T cells, which are glucose dependent in their activation, proliferation, and cytokine expression (IFN-γ) [34]. Guo et al. demonstrated that increased glycolysis in GBM induces T cell exhaustion through increased expression of checkpoint molecule PD-L1 [35]. Another study by Liang et al. calculated a glycolytic score for GBM tumors and found that higher glycolytic score was associated with increased malignancy and decreased survival with depression of multiple populations of immune cells including B cells, natural killer cells, and T cells. Furthermore, there was a strong association between higher glycolytic score and immunosuppressive TAM phenotype [36].

Pro-tumoral and anti-tumoral immune cells have different sensitivities and capacities to adapt to glucose depletion in the GBM TME. Pro-tumoral immune cells include M2-polarized TAMs and regulatory T cells (Tregs). These cells are better adapted to low glucose states as they utilize oxidative phosphorylation (OXPHOS) and fatty acid oxidation (FAO) preferentially to maintain long-term suppressive function [37]. These cells also possess more mitochondrial content and increased expression of PPARγ and CPT1a, allowing them to effectively metabolize lipids. In contrast, anti-tumor immune cells such as effector CD8^+^ T cells and M1-polarized TAMs are more susceptible to dysfunction in a glucose-depleted environment as they rely on aerobic glycolysis to support their proliferation, cytokine production, and cytotoxic functions [38]. Indeed, the GBM TME can affect the bioenergetic profile of anti-tumor CD8^+^ T cells through small molecules such as Meteorin-like (METRNL) [39] (Table 1). Jackson et al. showed that METRNL in the GBM TME can cause T cell mitochondrial depolarization and a metabolic shift away from oxidative phosphorylation towards glycolysis. The authors showed that METRNL exposure induces bioenergetic failure of T cells and causes CD8^+^ T cells to become hypofunctional, and METRNL downregulation improves the anti-tumor function of these CD8^+^ T cells.

Glucose starvation in T cells also inhibits mTOR signaling. mTOR is a critical regulator of glycolysis and anabolic growth in activated T cells [40] (Table 1). Upon T cell receptor stimulation, mTOR promotes glucose uptake to support T cell proliferation and effector function. However, in the nutrient-deprived GBM TME, mTOR activity is suppressed in T cells, which leads to decreased mitochondrial function, reduced granzyme B expression, ineffective tumor killing, and premature exhaustion [20]. The metabolic disequilibrium is further reinforced by VEGF and PGE2 secretion, which compromise local vascular perfusion and restrict nutrient delivery to immune cells, creating hypoxic immune “dead zones” in the tumor center [12,41,42].

Lactate is a key metabolic product of glycolysis that has a significant role in GBM metabolism and immunosuppression [43,44]. As a byproduct of glycolysis, lactate is exported from tumor cells into the TME by co-transport with hydrogen ions (H^+^). Thus, the release of lactate and H^+^ into the TME causes a lactate buildup and lowers extracellular pH. This accumulation of lactate and acidification of the TME induces M2 polarization of TAMs, facilitates expansion of Tregs via activation of anti-inflammatory gene programs, and alters monocarboxylate transporter function (MCTs) in immune cells to suppress the efflux capacity of these cells and further promote glycolytic depletion [1,13,17,18,45,46]. Extracellular lactate inhibits mitochondrial respiration in CD8^+^ T cells by blocking oxidative phosphorylation, causing mitochondrial depolarization, and generating reactive oxygen species [17,19]. Lactate blocks dendritic cell maturation via GPR81-dependent signaling, preventing antigen uptake and the expression of costimulatory molecules [47]. Elevated lactate has also been shown to induce epigenetic changes such as histone acetylation which promotes myeloid cell polarization toward a more anti-inflammatory M2 phenotype [48,49].

In GBM, it has been shown that elevated lactate levels can increase cell proliferation and migration in vitro and in vivo [50,51]. Several studies in solid tumors have evaluated the impact of alleviating acidity in the TME on immune cell function. For instance, Calcinotto et al. showed that lowering pH of cultured human and mouse T cells in vitro diminishes their efficacy while returning the pH back to normal alleviates these effects [52]. In a mouse model of B16-OVA-bearing mice, they found that proton pump therapy to abrogate TME acidity improved the efficacy of both dendritic cell vaccine therapy and adoptive T cell transfer therapy. Given that glioblastoma produces a significant amount of lactate and acidifies the TME, there is potential in studying this pathway in GBM as well and targeting this mechanism with combinatorial strategies (Table 1).

## 4. Amino Acid Metabolism and Immunosuppression

Whereas glucose deprivation inhibits immune cell effector function, glutamine deprivation changes the immune landscape in the TME [53]. GBM cells metabolize glutamine into α-ketoglutarate (α-KG) and utilize the metabolic intermediates generated in this process for a host of cell functions [54,55]. Mesenchymal GBM subtypes in particular rely heavily on glutamine, utilizing this critical metabolite to support the TCA cycle, nucleotide synthesis, and epigenetic modulation through α-KG–dependent dioxygenases [19,38,56]. Because tumor cells consume glutamine rapidly and deplete it from the TME, immune cells, such as dendritic cells and CD8^+^ T cells, are deprived of a key nutrient required for oxidative metabolism and histone demethylation, leading to defective antigen presentation and memory T cell generation [19,57]. Moreover, glutamine-deprived T cells secrete more anti-inflammatory cytokines such as IL-10 and TGF-β and less pro-inflammatory cytokines such as IFN-γ [38]. Glutamine deficiency also polarizes TAMs and MDSCs toward more immunosuppressive phenotypes and promotes Treg expansion [56,58].

**Figure 1:**
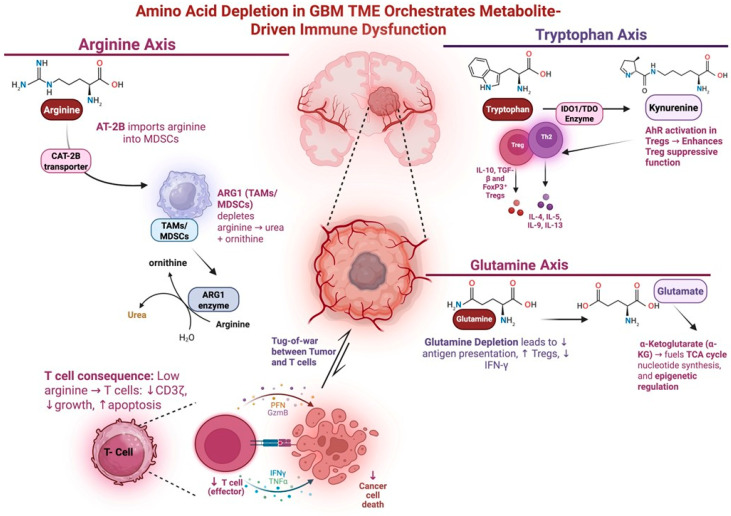
Amino acid depletion in the GBM TME promotes immune dysfunction. This schematic depicts how amino acid depletion in the GBM TME contributes to immunosuppression. Tumor cells consume critical amino acids such as glutamine, arginine, and tryptophan via overexpression of metabolic transporters and metabolic pathways (GLUT1/3, IDO1, ARG1), outcompete immune effector cells, and induce T cell dysfunction. In the arginine axis, AT-2B imports arginine into MDSCs and TAMs. This depletes arginine in the TME and leads to diminished Effector T cell growth and function. In the tryptophan axis, IDO converts tryptophan into kynurenine. Tryptophan depletion promotes Treg function and suppresses effector T cell function. In the glutamine axis, glutamine is taken up by tumor cells and converted to substrates the TCA cycle. Depletion of glutamine in the TME ledas to decreased antigen presentation and increased Treg function. Figure made with Biorender.com.

Depletion of vital amino acids such as tryptophan and arginine also plays a key role in GBM immunosuppression (Figure 1). Tryptophan depletion induces CD8^+^ T cell anergy and promotes Treg differentiation [59]. Elevated tryptophan metabolism also selectively promotes Treg proliferation while starving effector T cells of necessary amino acids [60,61]. Depletion of arginine triggers downregulation of CD3 and aberrant T-cell receptor signaling, leading to proliferative arrest and apoptosis [62]. During tryptophan or arginine scarcity, enzyme GCN2 activates in T cells and induces a stress response that halts protein synthesis and leads to proliferation arrest [63]. GBM tumors exploit this pathway by overexpressing enzymes such as IDO1 and arginase-1, allowing the tumor cells to rapidly consume tryptophan and arginine. Depletion of these amino acids initiates GCN2-mediated immune dysfunction and contributes to GBM immunosuppression in the TME.

IDO1 is an enzyme expressed in 90% of GBM tumors and has a crucial role in immunosuppression [64]. IDO1 and tryptophan-2,3-dioxygenase (TDO) metabolize tryptophan and have been implicated in immunosuppression across many cancers. IDO1 depletes tryptophan from the GBM TME depriving immune cells of this amino acid. IDO-mediated depletion of tryptophan in the GBM TME has been associated with decreased T cell growth and anergy through suppression of T cell activation [65]. Furthermore, IDO enzymatic activity results in the accumulation of kynurenine which itself suppresses effector T cells and supports Tregs by acting as an endogenous ligand for the aryl hydrocarbon receptor (AhR) [66]. Initially, GBM expression of IDO1 was thought to positively correlate with tumor grade mediated by the metabolic effects of IDO1; however, recent work has also uncovered non-metabolic IDO1-driven pro-tumoral effects. Non-enzymatic IDO activity was shown to increase expression of complement factor H and factor H-like protein 1 (FHL-1). Increased expression of these factors promotes Treg and MDSC longevity while decreasing overall animal survival in preclinical studies [64].

IDO emerged as a promising target due to its implication in multiple mechanisms of GBM immunosuppression. Preclinical studies found that inhibition of IDO1 reduces the number of immunosuppressive MDSCs present in culture [67]. Furthermore, IDO1 inhibition was found to synergize with radiation therapy and anti-PD-1 checkpoint blockade immunotherapy to increase survival in GBM only when IDO1 was inhibited on non-tumor cells [68]. In clinical trials combining IDO1 inhibition with anti-PDL1 inhibitors across various non-GBM solid tumor types showed no added benefit of IDO1 inhibition [69]. The failure of this combination in other solid tumors has dampened some interest in exploring synergy between IDO and other immunotherapy strategies [25] (Table 2). Nevertheless, it is important to consider the key role IDO plays in GBM immunosuppression through both metabolic and non-metabolic pathways as this presents a potential therapeutic avenue to exploit in future studies.

Another key target for immunotherapy under investigation is arginine metabolism. Many GBM cells are unable to synthesize their own arginine, so tumors rely on a subset of GBM cells that compensate for this deficiency by upregulating mechanisms of arginine acquisition from the environment, thus depleting this vital amino acid from the GBM TME [72]. Studies have explored arginine deprivation therapy which aims to deprive GBM of a reliable source of arginine. Hajji et al. demonstrated in a pre-clinical study that arginine-depleting agent ADI-PEG20 improves the radiation sensitivity and anti-tumoral immune cell infiltration of the tumor in an immunocompetent murine GBM model [73]. Feng et al. established a novel score system based on arginine metabolism genes and demonstrated that higher arginine deficiency in the tumor correlated with more immune cell infiltration [74].

The hoarding of arginine by tumor cells can deplete this amino acid in the TME and deprive immune cells, contributing to GBM immunosuppression. TAMs, which comprise up to 30% of a GBM tumor mass, can express a spectrum of phenotypes ranging from a pro-inflammatory M1 state to an anti-inflammatory M2 state, and this spectrum of TAM phenotypes is affected by GBM and TAM-derived exosomes [75]. Studies seeking to characterize the pro-tumoral effect of GBM-derived exosomes identified that these exosomes contain arginase-1 (ARG1). ARG1 is an enzyme that catalyzes the hydrolysis of L-arginine into urea and L-ornithine. This enzyme can be found on immunosuppressive TAMs and MDSCs in the TME of multiple tumor types [76]. High ARG1 expression is associated with poor prognosis in colorectal cancer and squamous cell carcinoma of the head and neck [77]. Similarly to IDO1-driven tryptophan depletion, ARG1-mediated arginine depletion impairs the metabolic fitness and differentiation of tumor infiltrating lymphocytes (TILs) [78]. Arginine is required for synthesis of the T cell receptor (TCR), and its depletion in the TME results in decreased TCR expression [79] as well as reduced cytokine secretion and T cell proliferation [80]. In a preclinical study, Azambuja et al. demonstrated in vitro that selective ARG1 inhibition reversed the pro-tumoral effects of ARG1+ exosomes secreted by immunosuppressive TAMs in GBM [81]. TAMs and ARG1 are not the only mediators of arginine depletion in the TME, as MDSCs can also deplete arginine via cationic amino acid transporter-2B (CAT-2B). In a murine models of renal cell carcinoma, it has been shown that MDSC-mediated arginine depletion in the TME drives TIL depletion [82]. The first phase I clinical trial of ARG1 inhibition in combination with anti-PD-1 demonstrated only limited anti-tumor activity when studied in numerous solid tumors including non-small-cell lung cancer, gastric cancer, renal cell cancer, melanoma, and urothelial carcinoma [77]. Studies, to date, have not explored the role of targeting arginine metabolism in combination with immunotherapy for GBM. However, given this pathway’s prominent role in immune function, it may be a relevant therapeutic window to further explore despite the initially poor findings in other solid tumors.

## 5. Sphingolipid Metabolism Affects Immune Cell Trafficking

**Figure 2:**
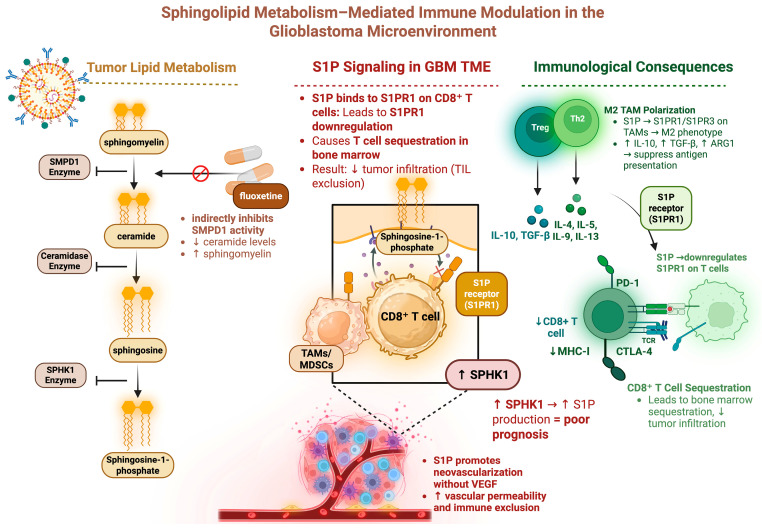
Sphingolipid metabolism can have numerous immunosuppressive consequences. This schematic depicts sphingolipid metabolism as it relates to GBM immunosuppression. S1P binding to CD8^+^ T cells increases T cell sequestration and reduces the number of T cells infiltrating the tumor. S1P binding to macrophages promotes M2 polarization which has an immunosuppressive effect. Figure made with Biorender.com.

Sphingolipid metabolism also plays a key role in GBM metabolism and immune resistance (Figure 2) [83]. The ceramide-S1P axis describes the balance between the pro-tumor sphingosine 1-phosphate (S1P) and its proapoptotic metabolic precursor ceramide. Ceramide is derived from sphingomyelin and is converted to the pro-apoptotic sphingosine. Phosphorylation of sphingosine by sphingosine kinase (SPHK1) results in the pro-tumor S1P, thus SPHK1 is a key enzyme in the generation of pro-tumoral S1P. Higher levels of S1P in human gliomas positively correlate with increasing tumor grade [84]. Early co-culture experiments of GBM tumor cells with endothelial cells demonstrated that blocking SPHK1 inhibited angiogenesis independent of VEGF signaling [84]. Later work uncovered that patients with high-SPHK1 GBM were found to have shorter survival [85]. Further investigation revealed that SPHK1 promotes GBM growth through multiple signaling pathways, including NF-*κ*B/IL-6/STAT3, JNK phosphorylation, and transcriptional activation of PTX3 [86]. SPHK1 overexpression resulted in increased proliferation, invasion, and metastasis, whereas its inhibition decreased tumorigenesis in an orthotopic glioma mouse model [86]. Recent work aimed at characterizing SPHK1 JAK2/STAT3 signaling has found that SPHK1 knockdown decreased tumor infiltration by anti-inflammatory immune cells [85].

In addition to SPHK1, the sphingomyelin pathway has other targets in GBM metabolism. Sphingomyelin phosphodiesterase 1 (SMPD1) mediates the conversion of sphingomyelin to ceramide and was found to be potently inhibited by the highly blood–brain barrier (BBB)-permeable SSRI fluoxetine. Bi et al. demonstrate that fluoxetine inhibition of SMPD1 in GBM was found to induce tumor cell death by reducing EGFR signaling and increasing lysosomal stress, resulting in complete tumor regression in mice [87]. Furthermore, they found in retrospective analysis of human GBM patients that increased survival was associated with fluoxetine administration but not other SSRI antidepressants.

Sphingomyelin metabolism in GBM can also alter GBM’s hypoxia-induced chemotherapy resistance. Given hypoxia-related EGFR signaling in GBM and sphingomyelin’s reduction in EGFR signaling, temozolomide (TMZ) in combination with an inhibitor of SPHK1 was assessed in a preclinical 3D spheroid GBM model by Sousa et al. This combination decreased GBM spheroid invasion and reduced the self-renewal capability of glioma stem cells (GSCs) especially under hypoxia, reversing hypoxia-induced resistance to chemotherapy [88]. Effective chemotherapy may provide more neoantigens for effective immunotherapy, so this supports the notion that sphingolipid metabolic modulation can be a promising target when considering combinatorial immunotherapy strategies.

Sphingolipid metabolism has also been implicated in affecting immune cell trafficking to the tumor. Chongsathidkiet et al. showed in a murine GBM model that T-cell sequestration in the bone marrow is associated with tumor-mediated loss of the T cell sphingosine-1-phosphate 1 (S1P1) receptor, one of five receptors that bind to the S1P ligand. Preventing the sequestration of this S1P1 receptor on T cells reversed the sequestration effect [89]. Interestingly, Guo et al. demonstrated that utilizing an S1P analog which binds to the S1P receptor family decreased recruitment of macrophages to the glioma TME and promoted a more M1 pro-inflammatory phenotype [90]. It is evident from these studies that the S1P1 receptor affects immune cell trafficking differently depending on the cell type involved. Leveraging these effects to increase TILs in the TME while decreasing immunosuppressive TAMs has the potential to significantly alter GBM TME-mediated immunosuppression.

## 6. Hypoxia in the GBM TME Contributes to Immune Resistance

The hypoxic TME of GBM has key metabolic implications that mediate tumor proliferation. GBM has significantly hypoxic regions which promote tumor growth, stemness, and immunosuppression [91,92]. Hypoxia induces HIF1α expression, which is known to increase CXCR4 in GBM [93], a key mediator of increased GSC invasion [94] and pro-tumor immune cell recruitment to the TME [95,96]. Furthermore, hypoxia-mediated secretion of IL-6 in GBM promotes autophagy which has been reported as an indicator of poor prognosis, where IL-6 blockade resulted in improved response to TMZ in xenograft models [97]. HIF1α/HIF2α–Sox2/Klf4 signaling occurs in hypoxic regions of GBM and promotes malignant progression via positive feedback of the EGFR–PI3K/AKT signaling pathway. HIF1α and HIF2α regulate Sox2 and Klf4 to increase stemness of the tumors and knockout of these genes resulted in chemo-sensitization to TMZ [98]. Hypoxia was found to increase tumorigenicity by direct metabolic reprogramming of GSCs by causing down regulation of DHFR and up-regulation of MAT2A, conferring GSCs the ability to proliferate independent of exogenous folate. Inhibition of MAT2A reversed the tumor initiating capability of GBM tumor-spheres in preclinical studies [99]. Hypoxia-driven chemotherapy resistance and tumor recurrence in GBM is in part due to increased activity of GSCs.

The adenosine pathway is a major immunometabolic signaling pathway in GBM upregulated by hypoxia. GBM immunosuppressive TAMs in the tumor core, compared to the tumor periphery, have increased glucose uptake and ATP synthesis [100]. GM-CSF cytokine secretion from tumor cells induces extracellular ATP (eATP) production by these TAMs. This eATP promotes glioma growth via the P2 × 7R receptor on glioma cells, suggesting that the eATP-P2 × 7R axis is a potential therapeutic pathway and highlights how GBM cells alter the metabolism of immune cells in the TME to promote tumor growth [100]. In addition to enhanced tumor signaling, CD39 and CD73 convert eATP to adenosine, a process known to be immunosuppressive [101]. Extracellular adenosine, through binding to the A2A adenosine receptor, inhibits anti-tumor TILs [102]. Hypoxia and TGFβ, a cytokine released by pro-tumor immune cells in the GBM TME [103], are the major drivers of the adenosine metabolic pathway that mediates pro-tumor proliferation, immune effector function, and immune exhaustion [104]. The efficacy of targeted inhibitors of this immunometabolic pro-tumor pathway in GBM are the focus of ongoing clinical trials. Specifically, CD73 was identified as a immunotherapeutic target to improve responses to immune checkpoint therapy in GBM [105] which is the focus of ongoing clinical trials in non-GBM advanced solid tumors [106].

## 7. The Relevance of Metabolic Alteration of the GBM TME on Immunotherapy Efficacy

Given the dismal prognosis of GBM with current standards of care, there has been significant interest in utilizing immunotherapy in GBM given its promising results in other cancers [2,107]. Unfortunately, clinical trials with GBM immunotherapy have been lackluster, and it is now being discovered that GBM employs numerous mechanisms of immune resistance including physical characteristics of the blood–brain barrier, intertumoral and intratumoral heterogeneity, and an immunosuppressive TME consisting of anti-inflammatory cytokines and immunosuppressive TAMs [108,109]. More recent literature suggests that GBM alters the metabolic landscape of the TME significantly to promote tumor growth and invasion while simultaneously suppressing anti-tumor immune activity [9,110,111] (Table 3). A better understanding of how metabolic alterations in the GBM TME contribute to immunosuppression can provide key insight into the lackluster results seen thus far with GBM immunotherapy strategies.

A hallmark of GBM immunosuppression is immune exhaustion, which is most evident in TILs, especially CD8^+^ T cells, which exhibit high and sustained expression of inhibitory checkpoint receptors such as PD-1, CTLA-4, TIM-3, and LAG-3 [112,113]. Activation of these checkpoint receptors inhibits TCR signaling, decreases interleukin-2 production, impairs granzyme/perforin-mediated cytotoxicity, and induce T cell senescence. Immune checkpoints are upregulated in GBM, so by inhibiting these immune checkpoints, researchers hoped to reverse TIL exhaustion. The co-expression of immune checkpoints linked to hierarchical dysfunction such that PD-1^+^TIM-3^+^LAG-3^+^ TILs are the most terminally exhausted phenotype with minimal reinvigoration capacity [112]. Given the observation of exhausted TILs in GBM, there have been numerous investigations into immune checkpoint blockades targeting various immune checkpoints including the PD-1/PD-L1 axis and CTLA-4. Immune checkpoint inhibitors showed significant promise in numerous preclinical studies but failed to translate to clinical benefit in phase 3 clinical trials [6,114,115]. Exhausted TILs in GBM have a unique TOX-regulated transcriptional signature that includes NR4A, EOMES, and BATF, which exert chromatin modulation and suppress effector gene loci [116]. Unlike melanoma or chronic viral infections, exhausted T cells in GBM notably do not have a TCF1^+^ stem-like progenitor population, which is the site of the response to checkpoint blockade [113]. This deficit could explain the lackluster response of PD-1 blockade in GBM patients and highlights a unique epigenetic barrier to immune reactivation in the TME. Despite the historically lackluster results of immune checkpoint inhibitors, there are still numerous trials underway exploring combinatorial strategies involving immune checkpoint blockade (Table 4). Of note, none of the studies presented in Table 4 below involve targeting specific GBM metabolic pathways, and this highlights the paucity of clinical research evaluating GBM immunotherapy in the context of GBM metabolism.

GBM vaccine therapy is another immunotherapy strategy under investigation, but unfortunately also demonstrated poor efficacy in clinical trials. An EGFRvIII tumor antigen-targeting peptide vaccine failed to demonstrate a survival benefit in a phase III clinical trial [117]. A limitation of cancer vaccines is that they rely on the host generating an effective immune response against the target antigen, and the therapy fails to be effective is the host immune response is not sufficient or cannot be sustained. Dendritic cell (DC) vaccination therapy may hold more promise in GBM as a recent phase III study published by Liau and colleagues showed promising statistically significant survival benefit in both primary and recurrent GBM patients [118]. For the cohort of primary GBM patients, mOS was 19.3 months with the DC vaccine versus 16.5 months in the placebo arm. For recurrent GBM patients, mOS was 13.3 months from relapse with vaccine treatment versus 7.8 months in the placebo arm.

Oncolytic virus immunotherapy has also been explored in GBM; however, it has failed to demonstrate a long-term anti-tumor T cell response [119]. Chimeric antigen receptor (CAR) T cell immunotherapy is a promising and currently evolving strategy that has demonstrated remarkable results in hematologic cancers such as leukemia and lymphoma [120,121,122,123]. CAR T cell therapy relies on ex vivo modification of the patient’s own T cells to express a designated tumor antigen and cellular domains that allow the T cell to remain constitutively active. However, a current limitation with this strategy in GBM is antigen loss and negative selection pressure leading to proliferation of a tumor cell population that lacks the CAR T cell antigen target [124]. Researchers are attempting to overcome this challenge by manufacturing newer generation CAR T cells that can target multiple tumor antigens simultaneously or release small molecules to alter the immunosuppressive milieu of the TME [125,126,127].

All of the immunotherapy strategies discussed above have failed to demonstrate a significant survival advantage in GBM to date. Of note, studies to date in GBM have not explored potential synergy between these immunotherapy strategies and therapies that target the numerous metabolic pathways utilized by GBM to promote immunosuppression. It is entirely possible that current immunotherapy strategies have been lackluster because these strategies rely on priming or activating T cells which may be unable to sustain a long-term effective anti-tumor effect due to an inability to survive in the harsh metabolic conditions in the GBM TME and effectively compete for necessary metabolites to sustain an effective anti-tumor response. It is likely that the metabolic conditions in the TME orchestrated by GBM may be significantly hampering the ability of these immunotherapy strategies to sustain long-term efficacy.

It is also important to consider immunotherapy strategies in the broader context of the current standard of care for glioblastoma, which also includes surgical resection. Surgical resection of the tumor core, including areas of hypoxia and necrosis, may improve survival not only by directly decreasing overall tumor burden but by also alleviating some of the immunosuppression induced by the necrotic, immunologically “cold” tumor core depleted of vital metabolites. Unfortunately, surgical resection is not able to cure GBM as microscopically invading cells often lead to recurrence despite adjuvant therapy. Indeed, it is important to note that given the significant tumoral heterogeneity of GBM, different tumor regions have been shown to have different metabolic profiles. Indeed, Baxter and colleagues found that 66 out of 168 metabolites were significantly different between glioma core and edge regions in paired samples from 27 patients [128]. The spatial differences in GBM metabolomics is relevant when considering surgical resection. Furthermore, there is a trade-off to surgical resection, as surgery has inherent risks and studies have shown that surgical resection of GBM can cause new permanent neurological deficits can actually diminish overall survival [129]. Additionally, surgical resection has diminishing returns for recurrent GBM, suggesting that surgically removing the immunosuppressive hypoxic and necrotic core of the tumor may slow down disease progression but ultimately is not enough to cure GBM.

## 8. Concluding Remarks

Despite the tremendous interest in investigating novel treatment strategies in GBM, thus far, these strategies have all been largely ineffective. Immunotherapy has found success in treating other solid tumors; however, this has not been translated to GBM due to multiple mechanisms of immune resistance. As our understanding of GBM pathophysiology and metabolism increases, it is becoming clearer that the same metabolic pathways utilized by the tumor for energy production and growth also play a role in immune resistance. This review article discusses several key GBM metabolic pathways, including (1) increased glycolysis depleting glucose and increasing lactate and acidity in the TME, depriving immune cells of a key source of energy and impairing their anti-tumor function; (2) metabolism of key amino acids such as glutamine, tryptophan, and arginine depleting these from the TME and preventing immune cells from maintaining sufficient access to essential building blocks; (3) sphingolipid metabolism affecting immune cell trafficking and function; and (4) hypoxia contributing to GBM chemotherapy resistance and promoting immune cell exhaustion. All of the key metabolic pathways discussed in this review directly affect immune cells in the TME. There is a paucity of preclinical and clinical studies evaluating synergy between targeting GBM metabolic pathways and classical immunotherapy strategies. Factoring in how to provide anti-tumor immune cells in the GBM TME with the nutrients they require to mount an effective anti-tumor response will be critical in improving GBM immunotherapy efficacy.

## Figures and Tables

**Table 1:** Glycolysis-Related Pathways Driving Immune Cell Exhaustion in GBM.

Sensor/Pathway	Trigger in GBM TME	Immune Effects	Therapeutic Implications
METRNL	Secreted by checkpoint-expressing tumor-infiltrating T cells	Bioenergetic failure of T cells and diminished effector function	METRNL inhibition may rescue anti-tumor T cell function
mTOR	Glucose deprivation, chronic stress	Impaired effector function, metabolic arrest	Consider mTOR agonists or T-cell metabolic reprogramming
Lactic acid	Key byproduct of glycolysis	Promotes M2 polarization of TAMs, Treg expansion	Alleviating lactate-driven acidity may improve T cell function and immunotherapy efficacy

**Table 2:** Select IDO Clinical Trials Including Subjects with GBM.

Trial ID	Study Start Date	Phase	Target	Other Results
NCT05106296	02/08/2022	1 (Recruiting)	IDO inhibitor	
NCT04049669	10/02/2019	2 (Recruiting)	IDO inhibitor	
NCT04047706	08/13/2019	1 (Active, not recruiting)	IDO1 inhibitor, anti-PD-1	Dose limiting toxicity of the combination is reversible hepatic transaminitis, phase 2/3 trial is approved [70]
NCT03707457	03/22/2019	1 (Terminated)	IDO1 inhibitor, anti-PD-1, anti-CTLA-4, anti-GITR	
NCT04587830	09/14/2020	2 (Recruiting)	ADI-PEG 20 (arginine depletion)	In newly diagnosed GBM subjects, peripheral arginine levels were suppressed with reciprocally elevated citrulline for 4–6 weeks, and preliminary OS was reported to be encouraging [71]

**Table 3:** Divergent metabolic programs of immune cells in the GBM TME.

Immune Cell Type	Metabolic Profile	Functional State in GBM	Immunological Role	Metabolic Dependencies
CD8^+^ Effector T cells	Aerobic glycolysis (Warburg effect)	Exhausted, hyporesponsive	Cytotoxicity, IFN-γ secretion	Glucose, mTOR, OXPHOS backup
Regulatory T cells (Tregs)	Fatty acid oxidation (FAO), OXPHOS	Expanded, suppressive	IL-10/TGF-β production, immune suppression	CPT1a, PPARγ, adenosine
M2-like TAMs	OXPHOS, lipid metabolism	Pro-tumoral, immunosuppressive	Angiogenesis, Treg recruitment, phagocytosis	FAO, lactate, AhR signaling
M1-like TAMs	Glycolytic, NO-driven metabolism	Rare, immunostimulatory	Antigen presentation, TNF/IL-12 production	Glycolysis, HIF-1α
Dendritic Cells (DCs)	Mixed; rely on glutamine for activation	Tolerogenic, immature	Poor antigen presentation, low costimulation	Glutamine, α-KG, mTOR

**Table 4:** Current clinical trials exploring combinatorial strategies involving immune checkpoint blockade.

Trial ID	Immune Checkpoint Target	Additional Treatments	Primary vs. Recurrent	Clinical Trial Phase
NCT06896110	Anti-PD-1	Azacitidine: disrupts DNA methylation, RNA processing, and protein synthesis	Recurrent	Phase I
NCT04145115	Anti-PD-1, Anti-CTLA-4	None	Recurrent	Phase II
NCT06325683	Anti-PD-1, Anti-LAG-3	Lomustine	Recurrent	Phase II
NCT03174197	Anti-PD-L1	Temozolomide and radiation	Primary	Phase I/II
NCT04977375	Anti-PD-1	Stereotactic radiation	Recurrent	Phase Ib/II
NCT02287428	Anti-PD-1	Neoantigen-based vaccine, radiation, temozolomide	Primary	Phase I
NCT05465954	Anti-PD-1	Recombinant IL-7	Recurrent	Phase II
NCT06558214	Anti-PD-1	TTFs, MRI-guided laser ablation	Recurrent	Phase II
NCT05084430	Anti-PD-1	Herpes oncolytic virus	Primary or Recurrent	Phase I/II
NCT05743595	Anti-PD-1	Neoantigen based personalized DNA vaccine	Primary	Phase I
NCT06672575	Bispecific antibody targeting PD-1 and VEGF	None	Recurrent	Phase I/II
NCT05039281	Anti-PD-L1	Tyrosine Kinase Inhibitor	Recurrent	Phase I/II
NCT04201873	Anti-PD-1	Dendritic cell vaccination	Recurrent	Phase I
NCT04003649	Anti-PD-1, Anti-CTLA-4	IL13Rα2-CAR T cells	Recurrent	Phase I
NCT06556563	Anti-PD-1	TTFs, Temozolomide	Primary	Phase III
NCT03277638	Anti-PD-1	Laser interstitial thermal therapy	Recurrent	Phase I/II
